# Sulfur-Induced Resistance against *Pseudomonas syringae* pv. *actinidiae* via Triggering Salicylic Acid Signaling Pathway in Kiwifruit

**DOI:** 10.3390/ijms222312710

**Published:** 2021-11-24

**Authors:** Zhuzhu Zhang, Youhua Long, Xianhui Yin, Sen Yang

**Affiliations:** 1 College of Agriculture, Guizhou University, Guiyang 550025, China; gs.zzzhang20@gzu.edu.cn; 2 Kiwifruit Engineering & Technology Research Center, Guizhou University, Guiyang 550025, China; yangsen2008812@163.com

**Keywords:** sulfur, induced resistance, canker of kiwifruit, salicylic acid, lignin, resistance mechanisms

## Abstract

Sulfur has been previously reported to modulate plant growth and exhibit significant anti-microbial activities. However, the mechanism underlying its diverse effects on plant pathogens has not been elucidated completely. The present study conducted the two-year field experiment of sulfur application to control kiwifruit canker from 2017 to 2018. For the first time, our study uncovered activation of plant disease resistance by salicylic acid after sulfur application in kiwifruit. The results indicated that when the sulfur concentration was 1.5–2.0 kg m^−^^3^, the induced effect of kiwifruit canker reached more than 70%. Meanwhile, a salicylic acid high lever was accompanied by the decline of jasmonic acid. Further analysis revealed the high expression of the defense gene, especially *AcPR-1*, which is a marker of the salicylic acid signaling pathway. Additionally, *AcICS1,* another critical gene of salicylic acid synthesis, was also highly expressed. All contributed to the synthesis of increasing salicylic acid content in kiwifruit leaves. Moreover, the first key lignin biosynthetic *AcPAL* gene was marked up-regulated. Thereafter, accumulation of lignin content in the kiwifruit stem and the higher deposition of lignin were visible in histochemical analysis. Moreover, the activity of the endochitinase activity of kiwifruit leaves increased significantly. We suggest that the sulfur-induced resistance against *Pseudomonas syringae* pv. *actinidiae* via salicylic activates systemic acquired resistance to enhance plant immune response in kiwifruit.

## 1. Introduction

The bacterial canker of kiwifruit is a devastating disease caused by *Pseudomonas syringae* pv. *actinidiae* (*Psa*). Since its initial discovery in Japan in 1984 [1], now this disease has been found in many countries, such as Italy [2], France [3], and New Zealand [4]. This disease was first discovered in the Hunan province, China, where it destroyed about 133.3 hm^2^ of kiwifruit orchards [5]. As the menace of the disease increased year after year, many kiwi-growing regions were infected with the disease, resulting in severe loss of yield worldwide.

Chemical control remains the primary option in the canker of kiwifruit during agricultural production. However, extensive chemical pesticides to control plant diseases can often generate resistance and cause pesticide-contaminated environments. Therefore, a novel and environmentally friendly method were explored in this study to control the canker of kiwifruit, and the underlying mechanism was elucidated. Sulfur is one of the 16 essential elements involved in plant growth and development, which can actively participate in the maintenance of the physiological function of the living organisms, regulate plant metabolism [6,7,8], and may also improve both the yield and the quality of kiwifruit [9,10]. Sulfur-induced resistance (SIR) denotes the reinforcement of natural resistance in the plants against pathogens by triggering different metabolic processes [11,12,13,14,15]. It can be directly or indirectly involved in plant disease resistance in various ways by participating in physiological and biochemical processes such as expression of plant cytoplasmic membrane structure, modulation of the protein metabolism, and regulation of enzyme activity [16,17,18,19].

Salicylic acid and jasmonic acid are essential signal molecules in the process of pathogen infection [20,21], which are involved in various aspects of regulating plant growth, environmental stress, and defense response to pathogens [22,23]. There is a close relationship between JA- and SA-mediated signal transduction pathway, synergy, and antagonism [24,25]. Exogenous chlorogenic acid, by activating the salicylic acid signaling pathway in peach, improved the fruit quality parameters of peach. Additionally, it enhanced the activities of pathogenesis-related proteins such as chitinase and glucanase [26]; *β*-1,3-glucanase and chitinase are essential pathogenesis-related proteins, and they can substantially destroy the cell wall of the pathogenic fungi and hydrolyze bacterial peptidoglycan, which plays an important role in pathogen defense [13,27,28,29]. When external factors stimulate plants, they can be induced and accumulated in a significant concentration. After chitosan was applied to tobacco, the expression of defense genes in tobacco was activated by SA, thereby inducing resistance to tobacco mosaic virus [30]. These SA-mediated responses also involve an increase in phenolic compounds such as lignin [31], which is a vital component of the secondary cell as it can enhance the structural strength of the host cell walls and form the basis of a self-defense strategy against the plant pathogen [32]. It was reported that lignin deposition could limit the pathogens to infected sites and prevent systemic infection [33].

In our previous study, we have reported that sulfur not only significantly promoted the growth, development, and fruit quality of kiwifruit but also induced the disease resistance of kiwifruits to *Psa*. The control effects in the greenhouse and field were observed to be more than 70% [9,10,34]. However, the mechanism of how sulfur can significantly improve disease resistance is still puzzling. In this study, the purpose was to evaluate the induce effect of sulfur on kiwifruit canker in the field and provide insight into the mechanisms involved. The results showed for the first time that the activation of the SA signaling pathway might play an essential role in the mechanism of sulfur-induced kiwifruit resistance to canker, mainly highly expressed *AcPR-1* and *AcPAL* genes. Thus, SAR is activated, resulting in increased lignin content and high disease-related proteins activity. This work is significant because sulfur is inexpensive and can be obtained easily. Understanding sulfur-induced resistance to kiwifruit canker and its possible physiological and molecular mechanisms will provide a new sustainable control strategy for kiwifruit canker, thus contributing to the development of new disease control approaches.

## 2. Results

### 2.1. Soil Treatment of Sulfur Enhances Kiwifruit against Pseudomonas syringae pv. actinidiae

**Figure 1:**
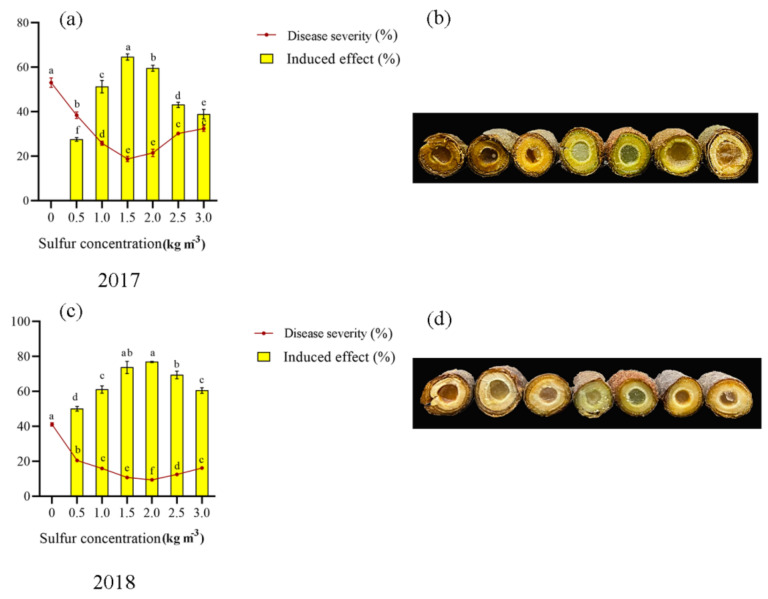
Induction effect on kiwifruit canker of sulfur. (**a**) Disease severity of kiwifruit canker disease and induce effect of sulfur in 2017; (**b**) Phenotypic map of kiwifruit stem canker disease after different sulfur treatments in 2017; (**c**) Disease severity of kiwifruit canker disease and induce effect of sulfur in 2018; (**d**) Phenotypic map of kiwifruit stem canker disease after different sulfur treatments in 2018. Note: Different lowercase letters indicate significant differences between different sulfur treatments at *p* < 0.05.

Sulfur has a significant induced effect on kiwifruit canker (Figure 1). In all sulfur concentrations, kiwifruit canker disease severity decreased significantly. The greatest decrease in the disease severity was observed for sulfur concentration of 1.5–2.0 kg m^−3^.

The first year (2017) and the second year (2018) were 63.70–64.40% and 74.16–74.91% lower than the control, respectively. The 1.5–2.0 kg m^−3^ sulfur concentration treatment had the best induction effect on kiwifruit canker, with 59.54–64.65% in the first year and 73.74–76.90% in the second year, which was significantly different from other treatments. It shows that sulfur application can reduce the disease severity of kiwifruit canker, inhibit the occurrence of the disease and improve its induction effect to control the occurrence of kiwifruit canker effectively. Compared with 2017, the induction effect of sulfur application on kiwifruit canker increased by 12.33% and 22.54% in 2018. The incidence of kiwifruit canker in 2017 corresponds to the phenotype shown in Figure 1a, while 2018 corresponds to the phenotype shown in Figure 1b.

### 2.2. Sulfur Improved Photosynthetic Characteristics of Kiwifruit

**Figure 2:**
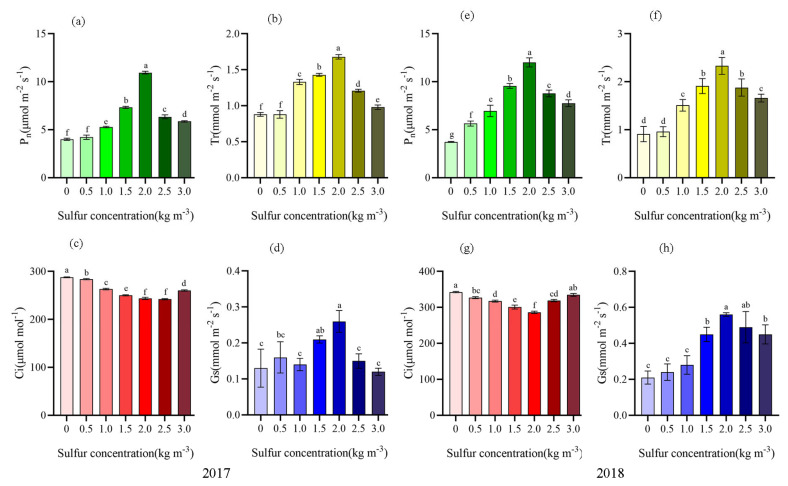
Effects of sulfur on photosynthetic characteristics of kiwifruit leaves. Different letters indicate significant difference between means at *p* < 0.05. Value are means ± SE (n = 3). Abbreviations: Pn, net photosynthesis rate; Tr, transpiration rate; Gs, stomatal conductance; and C_i_, intercellular CO_2_ concentration. (**a**) Net photosynthesis of kiwifruit leaves in 2017; (**b**) Transpiration rate of kiwifruit leaves in 2017; (**c**) Intercellular CO_2_ concentration of kiwifruit leaves in 2017; (**d**) Stomatal conductance of kiwifruit leaves in 2017; (**e**) Net photosynthesis of kiwifruit leaves in 2018; (**f**) Net photosynthesis of kiwifruit leaves in 2018; (**g**) Intercellular CO_2_ concentration of kiwifruit leaves in 2018; (**h**) Stomatal conductance of kiwifruit leaves in 2018.

It can be seen from Figure 2 that after sulfur application (2017 and 2018), the net photosynthetic rate, transpiration rate, and stomatal conductance of kiwifruit were significantly increased, and the change of intercellular CO_2_ concentration was opposite, showing a gradual downward trend (Figure 2). When the sulfur concentration was 2.0 kg m^−3^, Pn, Tr, and Gs reached the maximum. In 2017, compared with no sulfur application, it increased by 59. 73%, 74. 07%, and 50.00%, respectively. In 2018, it still maintained significant enhancements of 69.05%, 60.94%, and 62.50% (*p* < 0.05). It indicated that when the fertilization concentration was 2.0 kg m^−3^, the net photosynthetic rate and transpiration rate were increased by regulating stomatal conductance to promote CO_2_ exchange.

### 2.3. Effect of Sulfur on Chlorophyll Content in Kiwifruit Leaves

**Figure 3:**
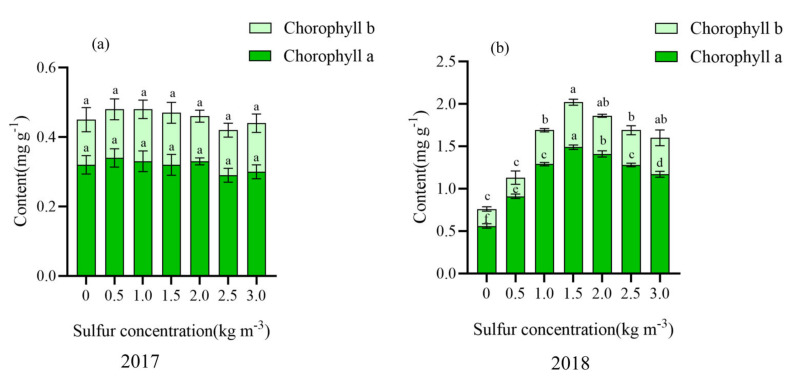
Effect of sulfur on chlorophyll content in kiwifruit leaves. Different letters indicate significant difference between means at *p* < 0.05. Value are means ± SE (n = 3). (**a**) Chlorophyll content of kiwifruit leaves in 2017; (**b**) Chlorophyll content of kiwifruit leaves in 2018.

Chlorophyll a and chlorophyll b did not change significantly in the first year of sulfur application (2017) but increased first and then decreased with the increase of sulfur concentration in the second year of sulfur application (2018). Moreover, the sulfur concentration was higher in the range of 1.5–2.0 kg m^−3^, increased by 60.28–62.42% and 55.56–62.26% compared with no sulfur (Figure 3).

### 2.4. Sulfur Activates the SA Signaling Pathways and Inhibits the JA Signaling Pathway

**Figure 4:**
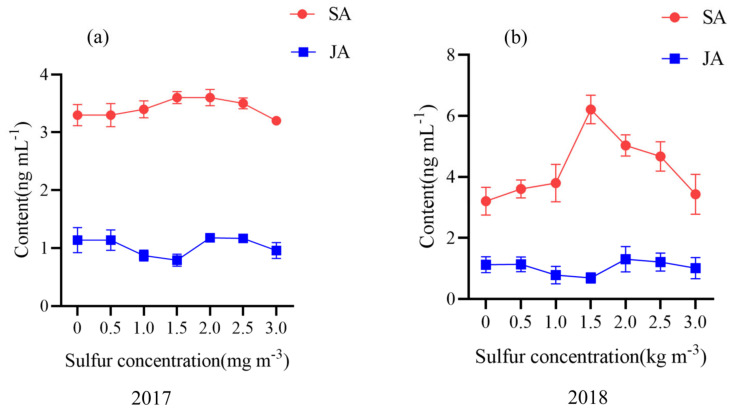
Effect of sulfur on salicylic acid and jasmonic acid content. Abbreviations: SA, salicylic acid; JA, jasmonic acid. Value are means ± SE (n = 3). (**a**) Contents of salicylic acid and jasmonic acid in kiwifruit leaves in 2017; (**b**) Contents of salicylic acid and jasmonic acid in kiwifruit leaves in 2018.

To clarify the changes in salicylic acid and jasmonic acid contents in kiwifruit after sulfur application and their relationship, we measured the contents of salicylic acid and jasmonic acid in kiwifruit leaves in 2017 and 2018 years of sulfur application (Figure 4). The response of salicylic acid to sulfur application was stronger than jasmonic acid, and the content of salicylic acid was higher than jasmonic acid for two consecutive years. When the sulfur concentration was 1.5 kg m^−3^, the content was high. In 2017 and 2018, salicylic acid content increased by 1.09 and 1.94 times, respectively, compared with no sulfur application. Surprisingly, there was a negative correlation between the change in SA content and JA content.

### 2.5. Expression of Resistance-Genes in Leaf and Stem of Kiwifruit under Sulfur Treatment

**Figure 5:**
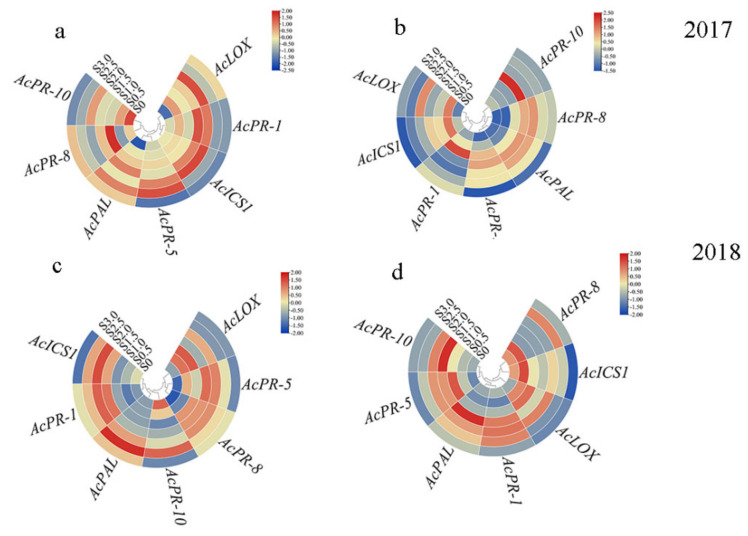
Expression pattern of defense-genes in leaves and stems of kiwifruit treated with sulfur. (**a**) Gene expression of kiwifruit leaves in 2017; (**b**) Gene expression of kiwifruit stem in 2017; (**c**) Gene expression in kiwifruit leaves in 2018; (**d**) Gene expression of kiwifruit stem in 2018. Note: The red and blue circles show up- and downregulated genes. The size of the circle was drawn to show the counts of differentially expressed genes.

In order to understand the ability of sulfur to activate the resistance gene of kiwifruit after two years of sulfur application, the expression of the resistance gene in kiwifruit leaves and stems was analyzed by gene expression circle diagram (Figure 5). In 2017, *AcICS1* gene and *AcPR-1* gene showed continuous high expression at sulfur concentration of 1.5–2.0 kg m^−3^ compared with no sulfur application. It increased by 26.67–36.54% and 42.86–49.21%, respectively (Figure 5a). *AcPAL* and *AcPR-8* were highly expressed in kiwifruit stem under this sulfur concentration range, and the gene expression levels were increased by 5.49–5.82 times and 5.35–5.77 times compared with those without sulfur application (Figure 5b). In 2018, *AcICS1*, *AcPR-1*, *AcPR-5,* and *AcPR-10* showed high expression levels at the sulfur application concentrations of 1.5–2.0 kg m^−3^, which were 1.07–1.13, 1.57–1.62, 1.55–1.59, and 4.91–5.00 times higher than without sulfur application, respectively (Figure 5c). *AcPAL*, *AcPR-1*, *AcPR-5,* and *AcPR-10* were highly expressed in kiwifruit stem, which was 3.85–4.54, 2.37–2.42, 1.79, and 7.86–11.19 times higher than those without sulfur application, respectively (Figure 5d).

### 2.6. Induction of Higher Lignin Accumulation and Deposition

**Figure 6:**
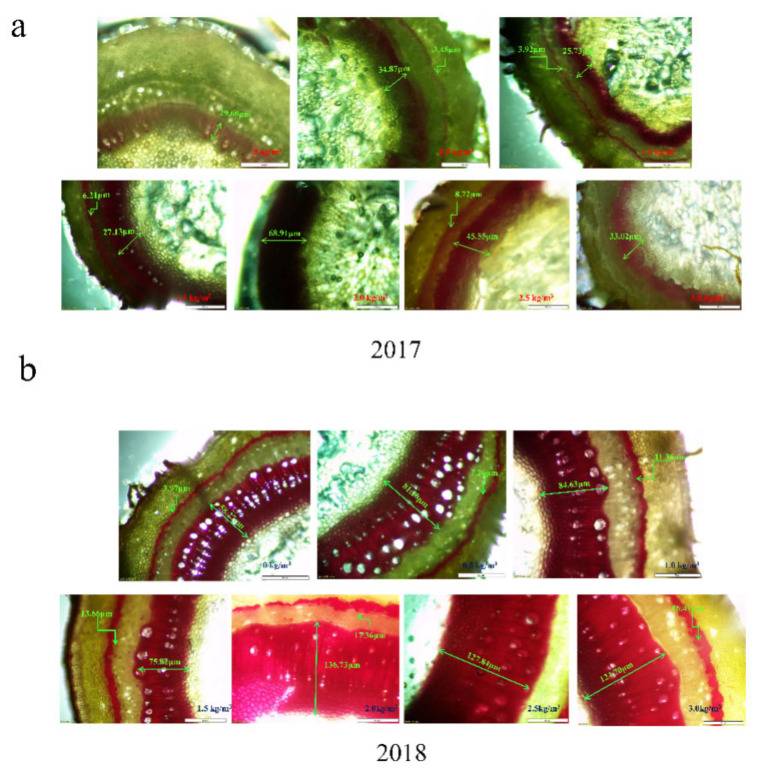
Effects of different sulfur concentrations on the lignin distribution and deposition in the stems of kiwifruit. (**a**) Lignin deposition in kiwifruit stem in 2017; (**b**) Lignin deposition in kiwifruit stem in 2018.

The results of the distribution and deposition of lignin in the kiwifruit stem have been shown in Figure 6, respectively. It was observed that lignin was mainly distributed in the two layers of kiwifruit stem; one layer was distributed between the epidermis and xylem of the kiwifruit stem (the outer layer), whereas the other layer was found between the xylem and pith of the kiwifruit stem (the inner layer). With increasing sulfur supply on kiwifruit, the lignin content in kiwifruit stem first increased rapidly and then declined (Figure 6), whereas the color change trend was light red−deep red−light red.

**Figure 7:**
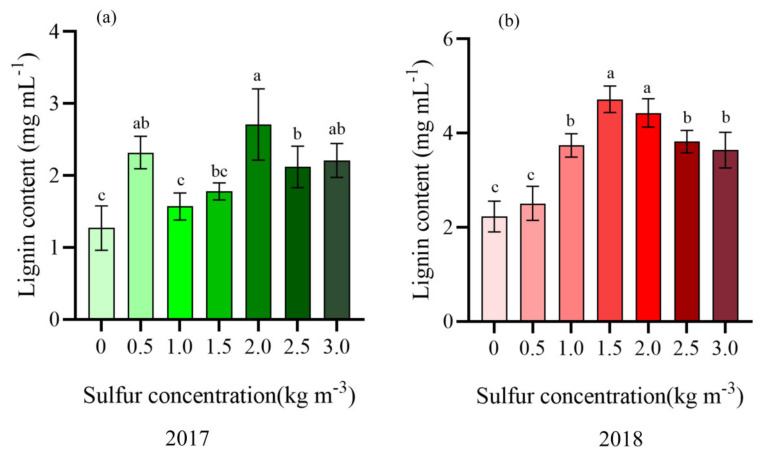
Effects of different sulfur concentration on lignin content in the stems of kiwifruit. Different letters indicate significant difference between means at *p <* 0.05. Value are means ± SE (n = 3). (**a**) Lignin content in kiwifruit stem in 2017; (**b**) Lignin content in kiwifruit stem in 2018.

In 2017, when the sulfur concentration was 1.5–2.0 kg m^−3^, the length of lignin staining bands in the inner layer was 27.35–68.91 μm (2.0 and 3.0 kg m^−3^ covered the outer layer due to the wide inner layer). There were significant effects upon 2.0 kg m^−3^ sulfur treatment, which resulted in a dark-red coloration. However, without sulfur treatment, the outer color was light, and almost no lignin was observed to be dyed red (Figure 6a). In 2018 (Figure 6b), the relationship between sulfur concentration and lignin content in stems was consistent with 2017. The application of sulfur at 1.5 kg m^−3^ was found to be 2.51 times higher than that without sulfur application (*p <* 0.05). The change in lignin deposition is consistent with the lignin content (Figure 7). Overall, the lignin distribution and deposition in 2018 were significantly better than in 2017 after sulfur application. The suitable sulfur concentration (1.5–2.0 kg m^−3^) can promote lignin accumulation in the kiwifruit stem. It may thus play an essential role as a potential physical barrier in sulfur-induced resistance of kiwifruit to canker.

### 2.7. Chitinase and ß-1,3-Glucanase Activity

**Figure 8:**
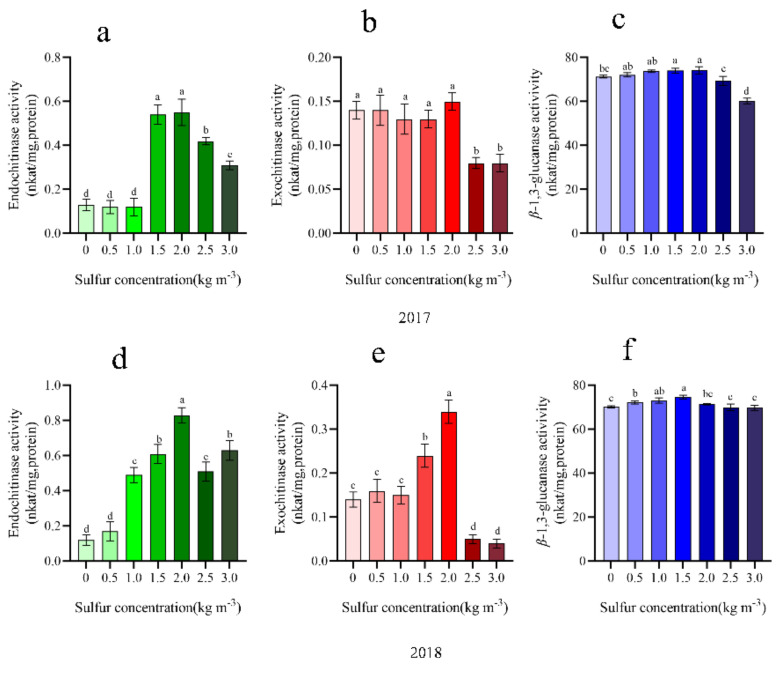
Effect of different sulfur treatments on chitinase and *β*-1,3-glucanase activities in the leaves of kiwifruit. Different letters indicate significant difference between means at *p <* 0.05. (**a**) Endochitinase activity in kiwifruit leaves in 2017; (**b**) Exochitinase activity in kiwifruit leaves in 2017; (**c**) *ß*-1,3-glucanase activity in kiwifruit leaves in 2017; (**d**) Endochitinase activity in kiwifruit leaves in 2018; (**e**) Exochitinase activity in kiwifruit leaves in 2018; (**f**) *ß*-1,3-glucanase activity in kiwifruit leaves in 2018.

**Figure 9:**
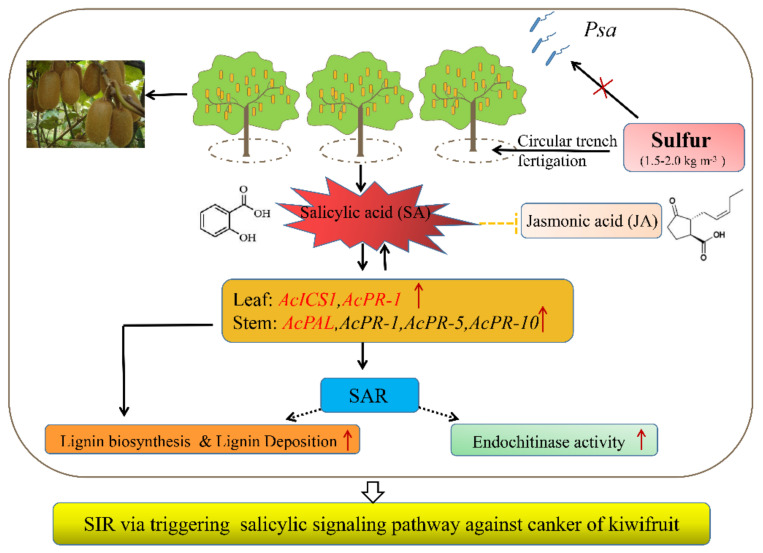
A proposed model describing how sulfur mediates salicylic acid and its pathway to regulate kiwifruit canker resistance was proposed. *PR-*1 and *ICS1* genes are involved in the activation of signals on SA and then activates systemic acquired resistance (SAR), contributing to the resistance of kiwifruit to *Pseudomonas syringae* pv. *actinidiae*.

Sulfur concentration in 1.5–2.0 kg m^−3^ can improve kiwifruit disease-related protease activity. In 2017, there was mainly an increase in the endochitinase activity, which was 75.93–76.36% higher than that without sulfur application (Figure 8a). In this range, there was no significant effect on chitinase and *β*-1,3-glucanaseenzymatic activity. In 2018, the activities of endochitinase showed the highest peak at 2.0 kg m^−3^ treatment compared with no sulfur treatment and increased by 85.54% (Figure 9), reaching a difference significant level (*p <* 0.05). However, when sulfur concentration was higher than 2.0 kg m^−3^, the enzyme activity demonstrated a marked declining trend. Moreover, sulfur also exhibited a significant benefit in favor of *β*-1,3-glucanase activity (Figure 8c,f). It was found that in response to increasing sulfur concentration, the activity first increases and then decreases gradually. Additionally, the application of 1.5 kg m^−3^ promoted *β*-1,3-glucanaseenzymatic activity quite efficiently and, as compared to no sulfur treatment, increased by 59.80%, with the difference being significant.

## 3. Discussion

Bacterial canker of kiwifruit caused by *Pseudomonas syringae* pv. *actinidiae* (*Psa*) has become the primary problem in the development of kiwifruit industry worldwide [35,36]. The disease often shows symptoms such as branch canker, leaf necrosis, and flower rot and has serious harm. It has expanded and spread in kiwifruit planting areas and lacks effective control measures. In particular, the characteristic kiwifruit series developed in many regions of China (such as the variety ‘Guichang’ in this study) are also highly susceptible, resulting in widespread destruction of orchards, serious economic losses, and serious setbacks to the enthusiasm of fruit farmers. Therefore, how to use various measures to effectively control kiwifruit bacterial canker has become the key to the development of the kiwifruit industry. At present, in the case that other prevention and control technologies have reached the bottleneck, the use of host plant disease resistance has become an important breakthrough in the prevention and control of canker disease, especially for kiwifruit with excellent traits that are also highly susceptible. Plant-induced disease resistance is an important research field of plant protection. It has become an effective supplement to chemical pesticides for controlling plant diseases due to its small dosage and good environmental compatibility [37], which is in line with the goal of reducing pesticide use and controlling pests and increasing production in China. Especially for some special diseases, there is no good chemical control method, through mineral nutrition regulation is a new method worth exploring [38].

Mineral nutrition not only promotes the healthy growth of plants but can also participate in plant disease resistance through diverse mechanisms [39,40,41,42]. Sulfur, as the fourth element needed by plants after nitrogen, phosphorus, and potassium, requires a large amount of nutrients [43], which can induce resistance to various plant diseases (SIR). At present, several mechanisms underlying SIR production in plants have been reported. These include (1) accumulation of cysteine, glutathione, sulfur-rich proteins, and the release of volatile S compounds, but cysteine and GSH play key roles in the process of disease resistance [44,45,46,47]; (2) regulation of the disease resistance signaling (salicylic, jasmonic acid, ethylene, abscisic acid, etc.) or the defense responses (ROS, stomatal opening, and closing), but these can vary significantly in the different disease systems [48,49,50]; and (3) production of the various plant antitoxins (such as glucosinolates and camalexin) [11,50]. Moreover, in the bacterial diseases caused by *P. syringae*, although no other sulfur control diseases and corresponding SIR reports have been documented previously, GSH could significantly induce salicylic signaling pathway independent of NPR1 and thereby increase the resistance of Arabidopsis to *P. syringae* [51]. Additionally, cysteine (rather than GSH) played a key role in the regulation of effector-triggered immunity of *P. syringae* [44].

In the early stage, it was observed that kiwifruit in the field was often in a state of sulfur deficiency. It was found that the application of an appropriate dose of sulfur could significantly reduce the incidence of kiwifruit canker (control effect > 70%). In addition, a continuous tracking following sulfur application was performed for two years, and the same control effect was found. However, sulfur had no direct bactericidal effect on *Psa* [34], thus indicating that SIR played an important role in kiwifruit canker resistance. However, its mechanism is still not fully unraveled.

At present, the studies on sulfur and plant resistance at home and abroad are primarily focused on fungal diseases. However, the possible effects on bacterial infections have not been extensively evaluated. In this study, the results showed that when the sulfur concentration was 1.5–2.0 kg m^−3^, the disease severity of kiwifruit canker decreased, and the induced effect was improved. In addition, sulfur also significantly improved physical and chemical parameters of kiwifruit fruit quality, which was consistent with Yin et al. [9]. Furthermore, the application of sulfur improved the net photosynthesis, transpiration rate, and stomatal conductance of kiwifruit leaves, which was consistent with the study [52]. This may be due to the amount of sulfur application once greater than 2.0 kg m^−3^, wherein the acidity of the soil is reduced, not suitable for the growth of kiwifruit plants, and kiwifruit plant growth potential is weak, resulting in kiwifruit plant resistance to canker.

The initial manifestation of plant infection with pathogens is rapid and localized death of infected cells, known as hypersensitive response (HR); the infected plants produce new resistance, which can be extended to the whole plant and usually become systemic acquired resistance (SAR). The significant sign of SAR is through salicylic acid signal transmission. Studies have shown that salicylic acid is mainly synthesized in plants through phenylalanine lyase (PAL) and isochorismate synthase (ICS)-mediated phenylalanine pathway. In this study, from 2017 to 2018, *AcICS1* in kiwifruit leaves and stems showed continuous high expression. However, there was no significant change in *AcPAL* gene expression (Figure 5). In addition, after two years (2017 and 2018) of sulfur application, the content of jasmonic acid in kiwifruit leaves fluctuated little, and the difference between the treatments was not significant, indicating that sulfur having little effect on the improvement of JA content in kiwifruit leaves was consistent with Lv et al. (2021), which indicated that after sulfur treatment, SA signal in kiwifruit was activated, while JA signal might be inhibited. Interestingly, high expression of salicylic acid synthesis marker gene *AcPR-1* was high expression. Therefore, we speculated that the high expression of the *AcICS1* gene might be mainly responsible for SA synthesis in stems and leaves of sulfur-treated kiwifruit. Our results are consistent with Jiao et al. [26]. *PRs* are divided into 17 families, such as pathogenesis-related gene 1 (*PR-1*) and exotic fruit-like protein (*PR-5*), which are often used to explain SA-mediated plant defense responses [53,54]. *PR-8* gene family encodes lysozyme and chitosanase, which kill Gram-positive bacteria. *PR-10* participates in plant defense responses through SA signal transduction pathway [55]. This study showed that when the sulfur concentration was 1.5–2.0 kg m^−3^, the gene expression levels of *AcPR-5*, *AcPR-8,* and *AcPR-10* in kiwifruit stems in 2018 were all activated (Figure 5d). A multitude of previous studies has shown that the production and deposition of lignin is one of the important mechanisms for inducing disease resistance [56,57]. When pathogens infect the plants, they often cause rapid lignification of the infected parts, which implies that lignin analogs are produced and can accumulate in the different parts such as the cell wall, intercellular layer, and the cytoplasm, which can prevent or delay the growth of pathogens and form a physical obstacle for the pathogen infection. Lignin biosynthesis can also play an important role in the long-distance transport of water, a process critical to plant survival [58]. The *PAL* gene is the key gene in lignin and phenolic compounds synthesis [59], and it is closely related to the expression of system acquired resistance [60]. In this study, when the sulfur concentration was 1.5–2.0 kg m^−3^, the *AcPAL* expression in stems was significantly increased in 2017 and 2018 (Figure 5), which was consistent with the increasing trend of lignin content (Figure 7). Therefore, the results of this experiment may be due to the significant increase in the *AcPAL* gene in the kiwifruit stem after sulfur application, which is involved in lignin synthesis and significantly increases the cell wall strength, thus hindering the invasion of *Psa* and disease. This is similar to the results obtained in the previous studies related to the control of pear fruit diseases by calcium [61], the improvement of rice sheath blight resistance by silicon [62], and the significant reduction in the incidence of soybean wilt, Fusarium, and Aspergillus by manganese [63]. Moreover, another mechanism by highlighted bacteria could spread in the plants was through the reproduction and secretion of bacterial fluid in the xylem, thereby blocking the ducts of plants to cause the disease [57,64]. A large number of studies have shown that the induction of plant resistance to pathogens may be attributed to the activation of highly coordinated defense-related enzymes such as CHI and GLU. Chitinase and *β*-1,3-glucanase are two important disease-related proteins. According to the different positions that chitinase can act on a potential substrate, it has been divided into endo- and exochitinases [44]. According to the different amino acid sequences, plant chitinases can be divided into four different categories. Among them, class III and class V chitinases can display additional lysozyme activity, which can effectively hydrolyze the alkaline enzyme of peptidase. They can potentially catalyze the hydrolysis of the *β*-1,4 glycosidic bond between peptidase N-acetylglucosamine and N-acetylcytic acid in the bacterial cell wall, destroy the peptidase scaffold, and expand as well as split the cells under the action of internal osmotic pressure, thereby resulting in the bacterial cracking [44]. In addition, in cotton, the expression of specific chitinase member genes was noted to be significantly correlated with the formation of secondary walls [65]. In this study, it was found that 1.5–2.0 kg m^−3^ sulfur treatment significantly increased the activities of chitinase exoenzymes and endoenzymes, and endoenzymes can contribute substantially to the chitinase activity (Figure 8a,d). An identical, fast increase trend in that range was observed. It can be speculated that sulfur may hydrolyze peptidoglycans in the pathogenic bacteria of kiwifruit canker, thereby disrupting the bacterial completion, in agreement with Gupta et al. [66].

Numerous studies have suggested that under the stress of exogenous substances, salicylic acid promotes the synthesis of lignin to improve the resistance to external pathogenic microorganisms [59,67]; it also increases disease-related protein activity, including chitinase and glucanase [61] and active resistance gene expression [26,30]. In this study, it was found that under sulfur treatment, activities of chitinase and *ß*-1,3-glucanase were induced to accumulate in kiwifruit leaves, mainly in the sulfur concentration of 1.5–2.0 kg m^−3^ range. In addition, it was also observed that the lignin content of the kiwifruit stem increased, and lignin deposition was obvious under this sulfur concentration range. However, whether the enhanced effect of sulfur on kiwifruit resistance to canker is due to the formation of a physical barrier, or if it can regulate the various metabolic processes between the plant and the pathogen, which can thereby induce the expression of disease resistance in the plants and the possible consequences of the synergistic effect of these two different aspects, need further analysis.

## 4. Materials and Methods

### 4.1. Materials

The sulfur fertilizer (S ≥ 95% purity) was purchased from Zhengan Agricultural Sci & Tech Co. Ltd., Shijiazhuang, China. The organic fertilizer (total nutrient content ≥ 4%, organic matter content ≥ 30%) was purchased from Guizhou Jilong Ecological Tec Co. Ltd., Guiyang, China.

### 4.2. Field Experiments

The field experiments were conducted for consecutive years (2017–2018) in a commercial orchard located in Xiuwen county, Guizhou province, China (26°45′34.4″N, 106°65′82.36″E,1168 m a.s.l) on 8 April. Field trials commenced on 24 December 2016; five kiwifruit plants were for one repetition and four repetitions were there for each sulfur concentration. The vines of the cultivar ‘Guichang’ were 18 years old, with 74 plants planted per 667 m^2^, including 68 female plants, trained in a T-bar trellis system. The trees received pollution-free planting cultivation techniques, and there were signs of infected *Pseudomonas syringae* pv. *actinidiae*, with the kiwifruit canker incidence reaching 76.7% in 2015 and 66.7% in 2016 [34].

The soil type in the experimental orchard is yellow soil, slightly alkaline (pH7.16). The soil samples were collected from a depth of 0–60 cm and analyzed [68]. Their concentrations are as follows: containing a total nitrogen content of 178 mg kg^−1^, available phosphorus content of 4.80 mg kg^−1^, available potassium content of 106.72 mg kg^−1^, available sulfur content of 4.80 mg kg^−1^. The sulfur powder and organic fertilizer were mixed homogeneously. The concentration of sulfur fertilizer has been listed in Table 1. We performed circular ditching (i.e., dig a rill outside the edge of the crown, 30–50 cm in width and 40 cm in depth; we used sulfur powder and organic fertilizer to make the base fertilizer mixed with topsoil to fill the ditch). Each experiment was performed in four different replicates.

### 4.3. Assessment of Disease Incidence: Disease Severity Index and Induction Effect

The incidence of kiwifruit bacterial canker disease was determined according to Long et al. [34]. The disease severity index was measured according to the rating scale of 1–6 after the incidence of canker in kiwifruit during the bleeding period (April) was investigated following sulfur application in the field for two years (2017–2018).

In the index, 1 = no disease (HR), 2 = less than 1/3 disease symptoms (R), 3 = 2/3 disease symptoms (MR), 4 = 1/2–3/4 disease symptoms (MS), 5 = more than 3/4 disease symptoms (S), and 6 = whole plants completely necrotic. Disease severity was calculated based on the formula = Σ (Number of plants infected with pathogenic bacteria × Representative value of corresponding class)/(Total number of investigated plants × Representative value of the highest-class) × 100. Induction effect (%) = (Disease severity of the control–Disease severity of the treatment)/Disease severity of the control × 100.

### 4.4. Photosynthesis Parameters and Fluorescence Parameters

The weather conditions met the requirements of clear sky and no wind, and we recorded the net photosynthesis rate (Pn), transpiration rate (Tr), stomatal conductance (Gs), and intercellular CO_2_ concentration (Ci) by LI-COR 6400 portable photosynthesis system (Li-COR, Huntington Beach, CA, USA).

### 4.5. Leaf Chlorophyll Content

The time to perform the analyses was 8 April 2017 and 2018, at 8:30 a.m. to 10:30 a.m., with randomly selected kiwifruit leaves with the same growth after different sulfur treatments. Kiwifruit leaves chlorophylls were isolated from acetone extraction technology. The fresh kiwifruit leaves (0.5 g) were cut into 1 cm slices and placed in 10 mL extract solution. The slices were soaked in the complete dark for about 20 h until the leaves became white. Two hundred μL pigment solution samples were taken, and the extraction solution was used as the control. The absorption value was determined by Tecan Infinite M200 of Switzerland, and the chlorophyll content was calculated according to previously described formulas [69].

### 4.6. Salicylic Acid (SA) and Jasmonic Acid (JA) Contents Measurements

Quantification of JA and SA in kiwifruit leaves was performed using a liquid chromatography-electrospray ionization tandem mass spectrometry (ESI) method according to De et al. [70] with slight modification. Chromatography conditions were modified by using a C18 column (Thermo, 250 × 4.6 mm), the temperature of the column oven was 28 °C, and the injection volume was 3.0 μL and set at a flow rate of 0.4 mL min^−1^. The mobile phases were 0.1% formic acid (mobile phase A) and 0.1% formic acid-acetonitrile solution (mobile phase B). Elution program (t min [%A: %B]) was: 0 min (95:5), 1.0 min (30:70), 2 min (30:70), 2.01 min (95:5), and 3 min (95:5). Mass spectrometry conditions were modified using a high-performance liquid chromatography-electrospray tandem mass spectrometry (LC-ESI-MS/MS) system. The ESI source operation parameters were as follows: the desolvation temperature was set at 650 °C with desolvation gas flow of 1000 L h^−^ and the cone gas at a flow rate of 30 L h^−1^. Data acquisition was accomplished with MassLynx V4.1 software (Waters Corporation) to deal with the base-peak ion current patterns of positive and negative ion modes. Meanwhile, the multiple reaction monitoring (MRM) mode was used for quantification.

### 4.7. RNA Extraction and Quantitative RT-PCR

Leaves and stems were collected at 8:30 a.m. to 10:30 a.m. randomly with the same growth after different sulfur treatments. The stems and leaves of kiwifruit were collected as RNA extraction materials in kiwifruit canker orchards. All RNA extraction materials were snap-frozen in liquid nitrogen and stored at −80 °C for standby use. A total of 0.5 g leaf and 0.5 g stem were ground in liquid nitrogen, respectively. Total RNA was extracted using the Plant Total RNA Isolation Kit (Huayueyang Biotechnology Co., LTD, Beijing, China) and synthesized into cDNA using RevertAid First-strand cDNA Synthesis Kit (Thermo Scientific, Waltham, MA, USA), the expression levels of *AcPR-1, AcPR-5, AcPR-8, AcPR-10, AcPAL, AcICS1, and AcLOX* were determined using *AcActin* as the reference gene for normalization. The gene-specific primers are provided in Table 2. Primers for quantitative real-time PCR (RT-qPCR) were designed by design net station (https://www.idtdna.com/Scitools/Applications/RealTimePCR/ access on 20 October 2021) on 15 May 2017 and 2018, respectively.

### 4.8. Measurement of Lignin Content and Histochemical Analysis

The measurement of lignin content was carried out based on the method from [71]. The stem was ground using liquid nitrogen, 0.1 g portion of the samples were weighed and collected in centrifuge tubes, then chlorophyll was extracted using 95% ethanol and centrifuged at 5000× *g* for 10 min, and the supernatant was removed. The deposition was washed three times with 2: 1(*v*/*v*) n-hexane: ethanol solution and centrifuged (5000× *g*, 10 min, 4 °C). The dried residue was then dissolved in 2.5 mL of acetyl bromide and glacial acetic acid (1:3, *v*/*v*) solution, heated in a water bath at 70 °C, and incubated for 30 min followed by quick cooling in an ice-water bath. The reaction was terminated after the addition of 0.9 mL 2 mol L^−1^ NaOH, 0.1mL 7.5 mol L^−1^ hydroxylamine hydrochloride 4 mL glacial acetic acid, then mixed as well as centrifuged (5000× *g*, 5 min, 4 °C), and 0.1mL of the supernatant was collected, which was diluted with 3.9 mL glacial acetic acid. The optical density (OD) value was measured using the UV-1800 ultraviolet spectrophotometer (Varioskan Flash Multimode Reader, Thermo Fisher Scientific, Waltham, MA, USA) at 280 nm. The results were expressed as g kg^−1^ based on the fresh weight. All the measurements were performed in triplicates. After two years of sulfur application in the kiwifruit field in 2017–2018, the new and old stems of kiwifruits were immediately brought back to the laboratory to visualize the potential distribution and deposition of lignin. The histochemical test of the kiwifruit stem was conducted as described previously by [72] with minor modifications. The ring slices of the kiwifruit stem were observed under a stereomicroscope (Nikon, Tokyo, Japan). Then, each stem was cut into two semi-thin and ultrathin sections with an LKB-2188 microtome hand-cut cross-sections, the sample was placed on the slide, and a drop of pure ethanol was added. Thereafter, incubation for 5 min was performed for phloroglucinol (Wiesner) staining, then the fresh sections were left for 1–3 min before being mounted with 50% HCl, and finally, lactophenol was added to obtain the slides. The observations were conducted under natural light, and the photographs of the stained slices were examined and photographed under a transmission electron microscope (JEM-1230, Japan). The glass slides with lactophenol served as control. The presence of lignin was confirmed by the appearance of the red stain on the tissues. The shades of red color represent the amount of lignin content.

### 4.9. Extraction and Assay of β-1,3-Glucanase and Chitinase

#### 4.9.1. Assay for Chitinase Activity

Extraction of enzyme solution: 0.5 g of kiwifruit leaves was taken after two years of sulfur application, liquid nitrogen was added and then ground to the powder form, followed by the addition of 7 mL sodium acetate buffer (pH = 5.0) homogenate. The mixture was centrifuged at 4 °C, 15,000× *g* for 15 min, after which the activity of extracellular chitinase and intracellular chitinase was analyzed by using the supernatant collected. The enzyme activity was measured based on a slightly modified method [73].

Standard curve of production: The different concentrations of N-acetyl glucosamine (Sigma Company, Burlington, MA, USA) at a volume of 1.5 mL were taken, and 2 mL of potassium ferricyanide solution was added, following which the mixture was boiled at 100 °C in a water bath for 15 min. The measured optical density in the distilled water was considered as a control. In contrast, the optical density decreased with increasing N-acetylamino glucose concentrations displaying a positive correlation, with n-acetyl glucosamine zero optical density being about 0.85. The standard curve was thereafter plotted with the difference of optical density (0.85 minus the density of sugar) as the coordinate and N-acetylglucosamine concentration as the abscissa.

Determination of endochitinase activity: The initial reaction solution used was the same as the assay of exochitinase. After being incubated at 37 °C for 2 h and centrifuged at 1000× *g* for 2 min, 1.5 mL supernatant was collected and added with 0.1 mL 3% (*w*/*v*) desalted snail enzyme and 0.15 mL 1 mol L^−1^ sodium phosphate buffer (pH = 7.1). The reaction solution was then placed in a constant temperature water bath at 37 °C for one hour to hydrolyze the various chitin fragments produced by chitin endonucleases. A reaction solution containing a substrate and an enzyme (heat-inactivated) was used as a control. The amount of N-acetylglucosamine produced was calculated by the method described above. One unit of 1 g N-acetylglucosamine per hour was produced from the decomposition of colloidal chitin.

Determination of exochitinase activity: 1.5 mL of colloidal chitin solution (containing 6 mg chitin) was absorbed, and 0.5 mL 50 mmol L^−1^ sodium acetate buffer solution (pH = 4.5) was added. The enzyme solution 0.4 mL and 0.1mL of 75 mol L^−1^ sodium azide solution was mixed well. After being kept at 37 °C for 2–4 h, 0.5 mL sodium borate buffer solution with a concentration of 0.8 mol L^−1^ (pH = 9.1) was added, and centrifugation at 1000× *g* for 5 min was performed. Thereafter, 1.5 mL supernatant was taken to measure the N-acetylglucosamine produced by the chitin extracellular enzyme. As the standard control, 1.5 mL of the same treated solution, including substrate and enzyme (heat-inactivated), was used. The optical density was measured at 585 nm, and the amount of N-acetylglucosamine produced was calculated from the standard curve based on the difference in the optical density (the standard control optical density minus the sample liquid optical density).

#### 4.9.2. Assay for β-1,3-Glucanase Activity

The enzyme activity was determined based on the method of [74]. Isochitinase was extracted from a crude enzyme solution. A total of 0.4 mL of 1 mg mL^−1^ laminarin (Sigma company), soluble in pH = 5.0, 50 tendency L^−1^ sodium acetate buffer, was taken and added to 0.1 mL enzyme liquid (diluted up to 10 times). Thereafter, the mixture was incubated for 15 min at 37 °C, and then immediately, 0.5 mL copper reagent was added, mixed, and incubated in a 100 °C water bath for 10 min. The mixture was thereafter cooled in ice water, and then 0.5 mL arsenic molybdate reagent was added. When the color turned to blue and distilled water was added after 3.5 mL, the colorimetric determination of absorbance was carried out at 540 nm with the use of glucose in the standard curve and enzyme as well as the substrate alone as a control. One Katal (Kat) is defined as the enzyme activity that can catalyze to produce 1 mole of glucose, and the unit of enzyme activity is nkat mg^−1^ protein.

## 5. Statistical Analysis

All the data shown are average values of at least three replicates with standard deviations. A one-way analysis of variance followed by Tukey’s HSD was performed. The data were analyzed using SPSS 18.0 (SPSS Inc., Chicago, IL, USA).

## 6. Conclusions

In summary, this study demonstrates that 1.5–2.0 kg m^−3^ sulfur concentration mixed with 10 kg m^−3^ organic fertilizer when applied to the soil to control the canker of kiwifruit displayed remarkable effects. The control efficiency of sulfur on bacterial canker disease was as high as over 70%, respectively. Furthermore, from the induced effects, photosynthetic traits, pathogenesis-related proteins (especially the endochitinase), and lignin content and expression of disease-resistant genes results, it is inferred that sulfur induces *Psa* resistance via the SA-dependent and JA-independent manner, implying that sulfur may be acting upstream of the SA and inhibits JA signaling pathways involved in plant defense, as described in Figure 9.

## Figures and Tables

**Table 1:** The concentration of sulfur treatment used in this study.

Number	Treatment
S_0_	Sulfur-deficiency+10 kg m^−3^ organic fertilizer (as a control)
S_1.0_	1.0 kg m^−3^ Sulfur powder +10 kg organic fertilizer
S_1.5_	1.5 kg m^−3^ Sulfur powder +10 kg organic fertilizer
S_2.0_	2.0 kg m^−3^ Sulfur powder +10 kg organic fertilizer
S_2.5_	2.5 kg m^−3^ Sulfur powder +10 kg organic fertilizer
S_3.0_	3.0 kg m^−3^ Sulfur powder +10 kg organic fertilizer

**Table 2:** Sequences for primers used in real-time fluorescent quantitative PCR.

Gene Name	Forward Primer	Reverse Primer
*AcActin*	CACCCTGTGCTGCTTACAGA	GAGAGAGAACGGCCTGAATG
*AcPR-1*	GCCCCCGGTAAGGTTTGT	CGAACCAAGACCCACTATTGC
*Ac* *PR-5*	TTCACCAACCTCAGTTCT	ATCGTAAGCGTAACTATAAGC
*Ac* *PR-8*	TTTGGATGGAATTGACTTTGACA	TTCTTGCCACGACTGCTATA
*Ac* *PR-10*	TGCTACACTTTAATTGAAGGC	TTGCTTGTCATCTTAGTAATCG
*Ac* *PAL*	CGGAGCAACACAACCAAGA	CCTGACATAGTGCGACTACATAG
*Ac* *ICS1*	AGGCGAGGCTTCTAATTG	ACAGCAAACTCACTCTCTC
*Ac* *LOX*	GGAGAAGCCATTGCCAAT	GGACGGTAATAAGTTGTGAAGTA

## Data Availability

All original articles used to support the findings of this study are available from the corresponding author upon request.

## Abbreviations

*Psa*	*Pseudomonas syringae* pv. *actinidiae*:
SA	Salicylic Acid
JA	Jasmonic Acid
SIR	Sulfur-induced resistance
SAR	Activates systemic acquired resistance
